# Associations between gut microbiome and circulating cytokines: a cross-sectional analysis in the FINRISK 2002 population cohort

**DOI:** 10.1186/s13099-025-00742-z

**Published:** 2025-08-26

**Authors:** Hassan Diab, Ville Langén, Li-Fang Yeo, Veikko Salomaa, Aki Havulinna, Leo Lahti, Katariina Pärnänen, Rob Knight, Sirpa Jalkanen, Marko Salmi, Teemu Niiranen, Joonatan Palmu

**Affiliations:** 1 Division of Medicine, Turku University Hospital, Turku, Finland; 2 Department of Geriatrics, Turku City Hospital and University of Turku, Turku, Finland; 3 Department of Internal Medicine, University of Turku, Turku, Finland; 4 Department of Public Health, Finnish Institute for Health and Welfare, Helsinki, Finland; 5 Institute for Molecular Medicine Finland, FIMM-HiLIFE, Helsinki, Finland; 6 Department of Computing, University of Turku, Turku, Finland; 7 Department of Pediatrics, University of California San Diego, La Jolla, San Diego, CA USA; 8 Center for Microbiome Innovation, Joan and Irwin Jacobs School of Engineering, University of California San Diego, La Jolla, San Diego, CA USA; 9 Department of Bioengineering, University of California San Diego, La Jolla, San Diego, CA USA; 10 Department of Computer Science and Engineering, University of California San Diego, La Jolla, San Diego, CA USA; 11 Halıcıoğlu Data Science Institute, University of California San Diego, La Jolla, San Diego, CA US; 12 MediCity Research Laboratory, University of Turku, Turku, Finland; 13 Institute of Biomedicine, University of Turku, Turku, Finland; 14 InFLAMES Research Flagship Centre, University of Turku, Turku, 20014 Finland

**Keywords:** Cytokines, Gut microbiome, C-reactive protein

## Abstract

**Background:**

A growing body of evidence suggests a relationship between gut microbiome and circulating cytokines, yet there is still a lack of large-scale population-based studies investigating gut microbiome-cytokine associations. In this cross-sectional study, we aimed at investigating the associations of gut microbiome (exposure variable) with 45 cytokines and C-reactive protein (CRP) (outcome variables) in the population-based FINRISK 2002 cohort (*N* = 2,398). Our analyses focused mainly on gut microbiome alpha diversity, beta diversity, differentially abundant taxa, and predicted functions. All statistical models were adjusted for age, sex, BMI, diabetes, and smoking.

**Results:**

Using linear modeling, we identified an inverse association of the gut microbial alpha diversity (Shannon index) with CRP (β=-0.062 ± 0.019/standard deviation (SD), False Discovery Rate adjusted p-value (FDR-P) = 0.025), interleukin-8 (IL-8) (β=-0.066 ± 0.021/SD, FDR-*P* = 0.025), and interferon-γ-inducible protein 10 (IP-10) (β=-0.063 ± 0.02/SD, FDR-*P* = 0.025). For beta diversity, linear modeling revealed that the first axis of Principal Component Analysis (PCA) describing the most strongly varying parts of the microbial community composition across population was inversely associated with CRP (β=-0.071 ± 0.019/SD, FDR-*P* = 0.008) and the second axis was inversely associated with macrophage inflammatory protein-1β (MIP-1B) (β=-0.082 ± 0.021/SD, FDR-*P* = 0.008), and monokine induced by interferon-γ (MIG) (β=-0.071 ± 0.019/SD, FDR-*P* = 0.008). The majority of the top taxa contributing to the first and second PCA axes belonged to class Bacilli (7/10) and class Gammaproteobacteria (9/10), respectively. In addition to this, we detected 8 significant associations of specific gut microbiome taxa (species-level) with cytokines and CRP using linear models. The majority of significant taxa belonged to class Clostridia_258483 (5/8) and class Bacteroidia (2/8). We did not detect any significant associations between species-specific predicted MetaCyc pathways (using all prevalent pathways) and cytokines or CRP. When analysis was limited to pathways associated with significant species only, we observed a positive association between purine synthesis predicted pathways in *B. thetaiotaomicron* and CRP.

**Conclusions:**

Taken together, these results show that CRP, MIP-1B, IL-8, and other cytokines are associated with gut microbial diversity and composition, as well as specific taxa. This could lay the groundwork for future experimental studies to assess the causality of these associations.

**Supplementary Information:**

The online version contains supplementary material available at 10.1186/s13099-025-00742-z.

## Background

Cytokines are soluble, low-molecular-weight protein messengers secreted from immune and stromal cells that mediate and coordinate the immune response [1]. Several factors, including age, gender, and annual seasonality have been shown to affect the production of cytokines in humans [2]. Specific gut microbial metabolic pathways including the degradation of dietary tryptophan to tryptophol have also been reported to modulate cytokine production and pathogen-induced immune responses in humans [3]. Oral administration of butyrate–a short-chain fatty acid produced by certain gut microbiota through the fermentation of dietary fibers–inhibited the production of pro-inflammatory cytokines in mouse models [4]. In a two-sample Mendelian randomization study between 196 gut microbiota and 41 inflammatory cytokines, putative causal associations were observed for phylum Euryarchaeota with interleukin-2 (IL-2), phylum Tenericutes and class Mollicutes with macrophage inflammatory protein-1α (MIP-1 A), class Bacilli with hepatocyte growth factor (HGF), order Enterobacteriales with monocyte chemoattractant protein-1 (MCP1), and genus *Lachnospiraceae NC2004 group* with tumor necrosis factor-related apoptosis inducing ligand (TRAIL) [5].

Previous studies have also suggested that the gut microbiome influences different disease entities through cytokine-mediated pathways. Monokine induced by interferon-γ (MIG) has been previously reported to mediate the casual association between *Ruminococcaceae UCG-*002 and diffuse large B-cell lymphoma [6]. In Mendelian randomization analysis, Firmicutes phylum decreased the risk of obstructive hydrocephalus and *Eubacterium ruminantium group* (genus) increased the risk of normal-pressure hydrocephalus, potentially through increased IL-17 A and decreased IL-27 levels, respectively [7]. Gut microbiota has been reported to contribute to the immune system maturation and intestinal homeostasis, and gut microbial dysbiosis can disrupt immune balance—particularly the Th17/Treg cell ratio—contributing to the development of inflammatory diseases [8].

The majority of the current evidence of the potential gut microbiome driven and cytokine-mediated disease pathways are based on selected patient samples highlighting the importance of studying these mechanisms in large-scale randomly selected population-based cohorts [6, 7, 9–13]. In this study, we studied the novel gut microbiome-cytokine associations using a panel of 45 circulating cytokines and a downstream inflammatory biomarker, C-reactive protein (CRP), with shallow shotgun sequenced gut metagenomics data in 2,398 participants aged over 50 years in the population-based FINRISK 2002 cohort.

## Methods

### Study sample

**Fig. 1:**
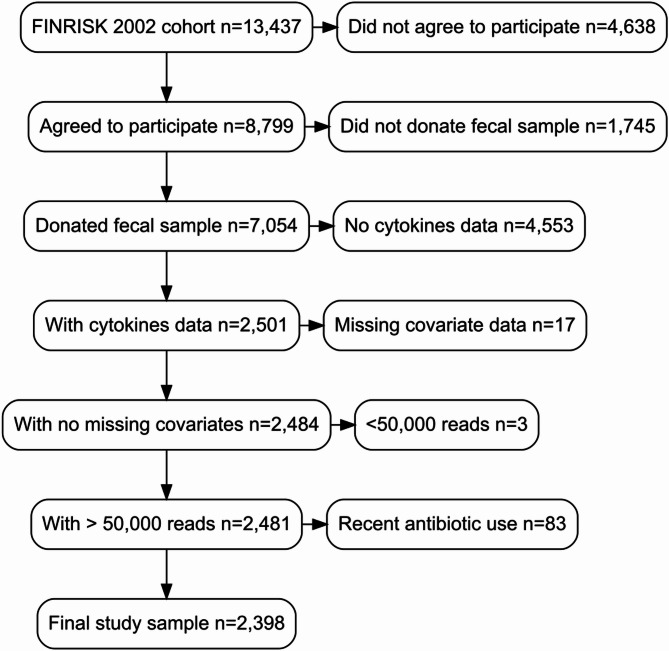
Participants included and excluded from the final study sample

The FINRISK 2002 cohort consists of individuals aged 25–74 years randomly selected from six different geographical areas of Finland [14]. In 2002, a random sample of 13,437 individuals was drawn from six geographic regions of Finland using the national population register. Altogether, 8,799 FINRISK 2002 individuals took part in the health examination. Fecal microbiome sequencing was performed successfully in 7,054 participants that donated fecal samples. From these participants, we excluded individuals with missing cytokine data (*n* = 4,553; due to budget constraints, only participants older than 50 years were selected for cytokine profiling [15]), missing covariate data (*n* = 17), metagenomics read count < 50,000 reads (*n* = 3), and antibiotic use within one month before baseline (*n* = 83), for a final study sample of 2,398 individuals who were included in this cross-sectional study (Fig. 1). None of the participants in the final study sample were pregnant.

### Questionnaire and health examination

The participants were asked to fill detailed questionnaire about their family history, diagnoses, use of medications, diet, functional capacity, and other health behaviors. All participants were invited to attend a health examination at local study centers, where they underwent anthropometric measurements and blood sample collection. Participants also received instructions for stool sample collection at home.

### Microbiome sequencing from stool samples

Stool samples were collected at home and mailed to the Finnish Institute for Health and Welfare overnight where they remained unprocessed at -20 °C. The microbiome analysis was performed with a whole-genome untargeted shallow shotgun metagenomic sequencing against mapped reference databases, as previously described [16]. In short, the sequencing of the samples was performed at the University of California San Diego in 2017, according to standard Earth Microbiome Project protocols [17]. Greengenes2 database was used to assign the taxonomy [18], and the data was imported in R TreeSummarizedExperiment container [19] for downstream analysis. Annotation of functional pathways abundances was performed using MetaCyc [20] database in the HUMAnN 3 pipeline [21].

### Measurements of cytokines and CRP

The measurement of baseline plasma levels of 48 circulating cytokines (pg/ml) was performed using Bio-Rad’s premixed Bio-Plex Pro Human Cytokine 27-plex Assay and 21-plex Assay, and Bio-Plex 200 reader with Bio-Plex 6.0 software [22]. The assays were performed according to manufacturer’s instructions, except that the amount of beads, detection antibodies, and streptavidin-phycoerythrin conjugate were used with 50% lower concentrations than recommended, as described earlier [23, 24]. CRP (mg/l) was determined by latex immunoassay (Abbott, Architect c8000). The Supplemental Table 1 shows the proportion of participants with detectable cytokine levels. Cytokines with more than 20% of no recorded data were dropped from the study (Supplemental Table 1); accordingly, interferon α-2 (IFN-A2), interleukin-15 (IL-15), and monocyte-chemotactic protein 3 (MCP-3) were removed from the current study.

The studied cytokines were interleukins (IL) IL-1 A, IL-1B, IL-1RA (receptor antagonist), IL-2, IL-2RA, IL-3, IL-4, IL-5, IL-6, IL-7, IL-8, IL-9, IL-10, IL-12-P40, IL-12-P70, IL-13, IL-16, IL-17, IL-18, monocyte chemoattractant protein-1/monocyte chemotactic and activating factor (MCP-1-MCAF, also known as C-C Motif Chemokine Ligand 2; CCL2), macrophage inflammatory protein-1α (MIP-1 A; CCL3), MIP-1B (CCL4), regulated upon activation normal T-cell expressed and secreted (RANTES; CCL5), eotaxin (CCL11), cutaneous T cell-attracting chemokine (CTACK; CCL27), growth-related oncogene-α (GROA; CXCL1), monokine induced by interferon-γ (MIG; CXCL9), interferon-γ-inducible protein 10 (IP-10; CXCL10), stromal derived factor-1α (SDF-1 A; CXCL12a), hepatocyte growth factor (HGF), leukemia inhibitory factor (LIF), stem cell factor (SCF), stem cell growth factor-β (SCGF-B), tumor necrosis factor-related apoptosis inducing ligand (TRAIL), platelet derived growth factor-BB (PDGF-BB), vascular endothelial growth factor (VEGF), macrophage colony-stimulating factor (M-CSF), fibroblast growth factor basic (FGF-BASIC), β-nerve growth factor (B-NGF), tumor necrosis factor-α (TNF-A), TNF-B, interferon-γ (IFN-G), granulocyte colony-stimulating factor (G-CSF), macrophage migration inhibitory factor (MIF), and granulocyte‐macrophage colony stimulating factor (GM-CSF).

### Variable definitions

BMI was measured in kg/m^2^. Smoking was defined as self-reported current daily smoking. Diabetes was defined as self-reported diabetes, a nationwide Care Register for Health Care-based diagnosis for diabetes (International Classification of Diseases, Tenth Revision [ICD‐10], codes E10‐E14 or ICD‐8/ICD‐9 code 250), a drug purchase related to diabetes (a drug with a code starting with Anatomical Therapeutic Chemical classification code A10), or a special reimbursement code for diabetes medications in the nationwide Drug Reimbursement Register.

### Statistical methods

Concentrations of 45 circulating cytokines and CRP were used as outcome variables. Cytokine observations below or above the detection limit were extrapolated towards the asymptote of the five-parameter logistic calibration curves [23]. Based on visual inspection, fluorescence intensity at the lower and upper asymptote were used to estimate the cytokine concentrations outside detection limits [25]. Because the calibration curves were plate-specific, it was possible to obtain plate-specific estimates for the observations outside the detection limits. We did not impute unmeasured cytokine concentrations. For CRP, values below detection level (less than 0.1 mg/L) were replaced by minimum detectable CRP value divided by two (0.05). Log-transformed and unit standard deviation scaled cytokine and CRP values were used in all the analyses except for ANCOM-BC2 in which log-transformed non-scaled cytokine and CRP values were used.

Alpha diversity was defined using Shannon index at species-level with mia R/Bioconductor package 1.15.21 [26]. For beta diversity, Principal Component Analysis (PCA) axes were calculated at species-level, using centered log-ratio (CLR) transformed microbial abundances [27]. CLR transformation was applied to relative abundance values and pseudocounts were used for zero values. For taxa-level differential abundance analyses, we used common taxa that were required to have prevalence over 5% in the sample population with a relative microbial abundance over 0.1%. For pathway analyses, we used common predicted MetaCyc pathways [20] that were required to have prevalence of at least 10% in the sample population. We used a higher prevalence threshold for pathways analyses due to the high number of predicted pathways. Analysis was also performed with pathways that were associated with significant taxa only.

The associations of the cytokines and CRP with (1) alpha diversity, (2) PCA (beta diversity) axes, (3) common taxa, and (4) common functional pathways were assessed using multivariable-adjusted linear regression models. The contribution of distinct taxa to PCA axes was determined by the coefficients of the eigenvectors that define each principal component. Differential abundance analyses of individual taxa were performed using CLR transformed microbial abundances. As a sensitivity analysis, the differential abundance analyses were also performed using Analysis of Compositions of Microbiomes with Bias Correction (ANCOM-BC2) [28]. The functional pathway counts were (1) dichotomized (present vs. absent) or (2) inverse-rank normalized due to the highly sparse and zero-enriched nature of the pathway data. All models were adjusted for age, sex, BMI, diabetes, and smoking. To check for the robustness of the results, we assessed the significance of the detected species-cytokine (and CRP) associations in subgroups of age, sex, BMI, and geographical area and also included additional covariates (including the use of proton pump inhibitors (PPIs), physical activity, alcohol consumption, income level, education level, and geographical area) to the main linear models (as a sensitivity analysis). We also compared the detected associations between diabetic and non-diabetic participants.

R version 4.4.1 was used for all statistical analyses. P-values were corrected for multiple testing using False Discovery Rate (FDR-P; Benjamini–Hochberg correction). FDR-*P* < 0.05 were deemed statistically significant.

## Results

The mean age of the participants was 60.1 ± 5.93 years and 48.2% were men (Table 1). A description of the study sample characteristics, stratified by geographical area, is available in Supplemental Table 2. The mean concentrations of the cytokines are listed in Supplemental Table 3. The common taxa included 268 species.

**Table 1:** Characteristics of the study sample

Characteristic	Mean (SD) / *N* (%)
N	2398
Age, year (SD)	60.1 (5.93)
Men	1157 (48.2%)
BMI (SD)	28.1 (4.50)
Diabetes	160 (6.7%)
Smoking	401 (16.7%)

SD, standard deviation; BMI, body mass index

### Alpha and beta diversity

**Fig. 2:**
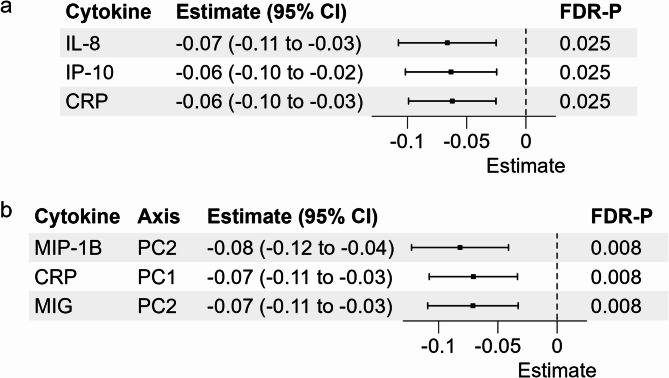
Associations of alpha diversity **(a)** and beta diversity **(b)** with cytokines and CRP, calculated using multivariable-adjusted linear regression models. Alpha diversity was defined using Shannon index at species-level. For beta diversity, PCA axes were calculated at species-level using CLR transformed microbial abundances. Estimates are reported per 1-SD increment. CI, confidence interval; FDR, false discovery rate; IL-8, interleukin-8; IP-10, interferon-γ-inducible protein 10; CRP, C-reactive protein; MIP-1B, macrophage inflammatory protein-1β; MIG, monokine induced by interferon-γ; PC, principal component

**Fig. 3:**
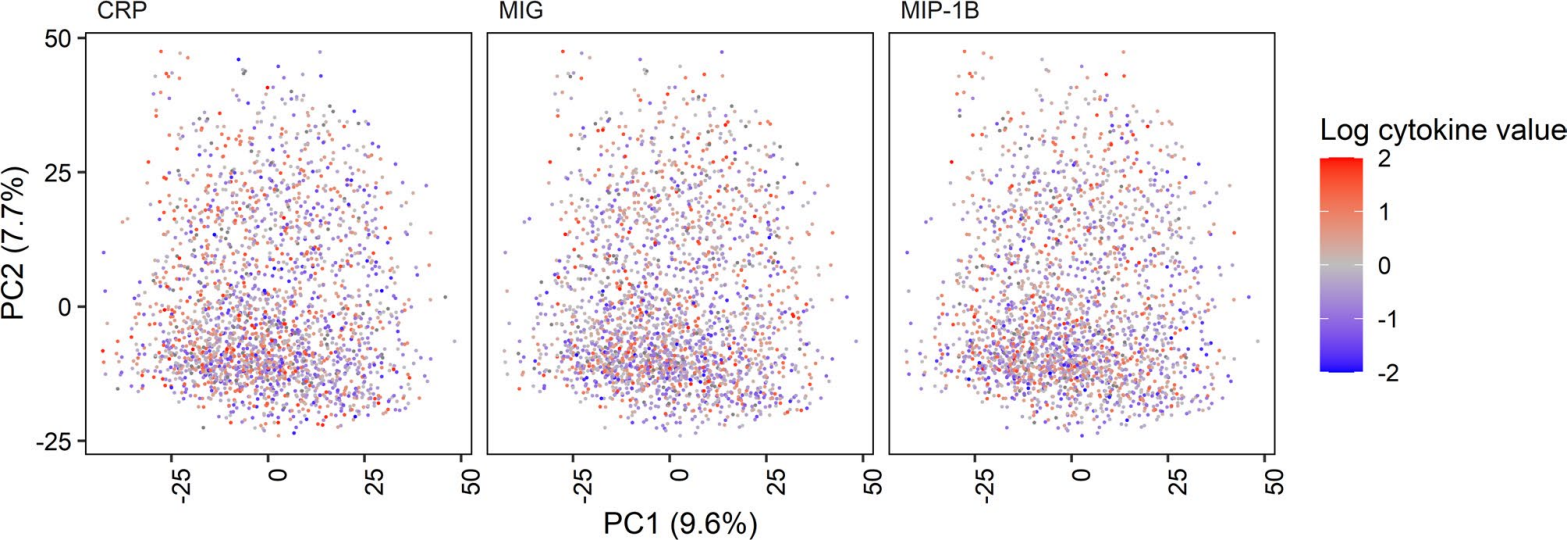
The first two PCA axes calculated at the level of microbial species. The concentrations (log-transformed, scaled) of the cytokines that were significantly associated with the first two principal components are illustrated. PC, principal component; CRP, C-reactive protein; MIG, monokine induced by interferon-γ; MIP-1B, macrophage inflammatory protein-1β. 4.4% of log-transformed cytokine values fall outside the selected color-coded range

Alpha diversity (Shannon index) was inversely associated with CRP (β=-0.062 ± 0.019/standard deviation (SD), FDR-*P* = 0.025), IL-8 (β=-0.066 ± 0.021/SD, FDR-*P* = 0.025) and IP-10 (β=-0.063 ± 0.02/SD, FDR-*P* = 0.025) (Fig. 2a, Supplemental Table 4). The first PCA axis was inversely associated with CRP (β=-0.071 ± 0.019/SD, FDR-*P* = 0.008) and the second PCA axis was inversely associated with MIP-1B (β=-0.082 ± 0.021/SD, FDR-*P* = 0.008) and MIG (β=-0.071 ± 0.019/SD, FDR-*P* = 0.008) (Figs. 2b and 3, Supplemental Table 5). The top ten species contributing to the first two principal components are shown in Supplemental Fig. 1; the majority of the top taxa contributing to the first and second PCA axes belonged to class Bacilli (7/10) and class Gammaproteobacteria (9/10), respectively (Supplemental Fig. 1, Supplemental Table 6).

### Differential abundance analysis

**Fig. 4:**
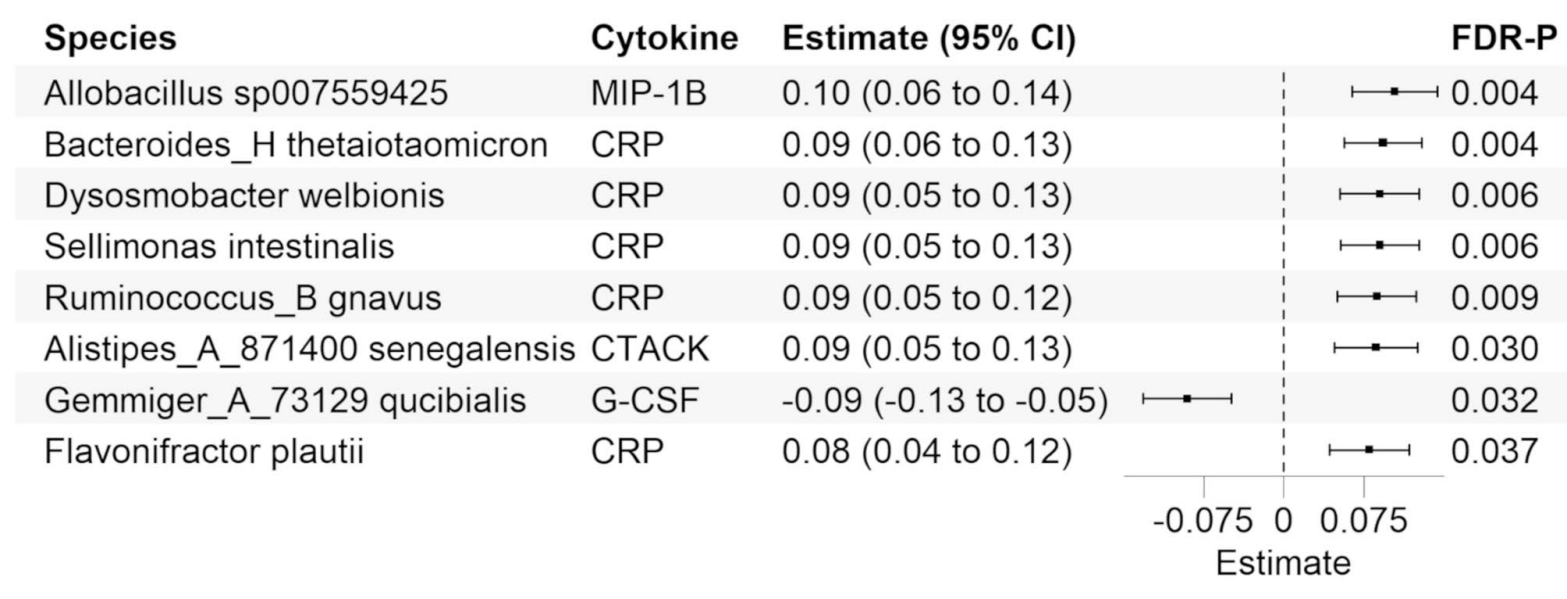
Species significantly associated with cytokines and CRP concentrations and detected using multivariable-adjusted linear regression models. Estimates are reported per 1-SD increment. CI, confidence interval; FDR, false discovery rate; MIP-1B, macrophage inflammatory protein-1β; CRP, C-reactive protein; CTACK, cutaneous T cell-attracting chemokine; G-CSF, granulocyte colony-stimulating factor

Analysis of differentially abundant species revealed a positive association between *Allobacillus sp007559425* and MIP-1B (β = 0.104 ± 0.020/SD, FDR-*P* = 0.004), an inverse association between *Gemmiger_A_73129 qucibialis* and G-CSF (β=-0.091 ± 0.021/SD, FDR-*P* = 0.032) and a positive association between *Alistipes_A_871400 senegalensis* and CTACK (β = 0.087 ± 0.02/SD, FDR-*P* = 0.03) (Fig. 4, Supplemental Fig. 2, Supplemental Table 7). In addition, *Bacteroides_H thetaiotaomicron* (β = 0.093 ± 0.019/SD, FDR-*P* = 0.004), *Dysosmobacter welbionis* (β = 0.09 ± 0.019/SD, FDR-*P* = 0.006), *Sellimonas intestinalis* (β = 0.09 ± 0.019/SD, FDR-*P* = 0.006), *Ruminococcus_B gnavus* (β = 0.087 ± 0.019/SD, FDR-*P* = 0.009) and *Flavonifractor plautii* (β = 0.08 ± 0.019/SD, FDR-*P* = 0.037) were all positively associated with CRP (Fig. 4). Most of the species-cytokine (and CRP) associations remained significant across subgroups of age, sex, BMI, and geographical area (Supplemental Table 8). Additionally, the direction of the estimates of the detected associations remained consistent between diabetic and non-diabetic participants, however, and due to the small number of diabetic individuals (*n* = 160), these associations were not significant in diabetic individuals (Supplemental Table 8). The addition of more covariates (including the use of proton pump inhibitors, physical activity, alcohol consumption, income level, education level, and geographical area) did not have a major effect on the detected associations (Supplemental Table 9). Four out of the five significant species-CRP associations identified by linear regression were replicated with ANCOM-BC2 (Supplemental Table 10).

### Functional analysis

No significant association was detected between species-specific predicted MetaCyc pathways (using all prevalent pathways) and cytokines or CRP using dichotomized (Supplemental Table 11) or inverse-rank normalized count data (Supplemental Table 12). When analysis was performed only in pathways associated with the significant taxa, we observed positive associations between *B. thetaiotaomicron* predicted pathways and CRP using inverse-rank normalized count data (5 positive associations) and dichotomized count data (one positive association) (Supplemental Fig. 3, Supplemental Tables 13–14). The majority of these pathways were related to purine synthesis.

## Discussion

In the present study, we investigated the association of gut microbiome with 45 circulating cytokines and CRP in a large population-based study. We detected 8 significant associations of specific gut microbiome taxa (species-level) with cytokines and CRP using linear models. The significant taxa belonged to class Clostridia_258483 (4 positive associations and 1 inverse association), class Bacteroidia (2 positive associations) and class Bacilli (1 positive association). Additionally, we identified an inverse association of IL-8, CRP, and IP-10 with gut microbiome alpha diversity, while for microbial beta diversity, we observed an inverse association between CRP and the first PCA axis (driven mainly by taxa belonging to class Bacilli) and an inverse association of MIP-1B and MIG with the second PCA axis (driven mainly by taxa belonging to class Gammaproteobacteria). We did not detect any significant associations between species-specific predicted MetaCyc pathways (using all prevalent pathways) and cytokines or CRP. When we limited the analysis to pathways associated with significant taxa only, we observed 5 positive associations between *B. thetaiotaomicron* pathways and CRP, the majority of which were related to purine synthesis.

Linear regression revealed a positive association between MIP-1B and *A. sp007559425*. MIP-1B was also linked to beta diversity as revealed by PCA. MIP-1B is a chemokine involved in the recruitment of immune cells and its expression is induced by bacterial endotoxins [29, 30]. The studies on the association of MIP-1B with gut microbiome taxa are currently limited. Nevertheless, a previous work aimed at studying the relationship between gut microbiome changes and cytokines in patients with post-COVID syndrome reported an inverse association between MIP-1B and *Eubacterium hallii* [31]. Moreover, a previous animal study has reported a significant role of MIP-1B in shaping the gut microbiome composition in high-fat-diet-induced diabetes mellitus mice [32]. It was observed that after MIP-1B inhibition, the relative abundance of family *Muribaculaceae* was reduced, while that of family *Atopobiaceae* increased. More studies are required to understand the relationship between gut microbiome and MIP-1B.

We also observed a positive association of *A. senegalensis* with CTACK, a chemokine produced by skin cells and functions in attracting memory T cells [33]. Recent Mendelian Randomization studies have shown that *A. senegalensis* is positively linked to longevity [34], and inversely associated with marginal zone lymphoma [35] and cervical disc disorders [36]. Moreover, animal studies have identified a positive effect of *A. senegalensis* on locomotion in mice [37]. Our study adds more evidence to the importance of *A. senegalensis* as a member of the gut microbiome, and its possible connection to the immune system, which requires further investigation.

CRP was positively associated with *B. thetaiotaomicron*, *D. welbionis*, *S. intestinalis*, *R. gnavus*, and *F. plautii* as detected using linear regression. Most of the detected associations (4/5) were replicated when analyses were performed using ANCOM-BC2. Chen et al. detected an interaction between CRP and genus *Ruminococcus* with suggestive significance for Generalized Anxiety Disorder-7 score, a measure of anxiety [38]. Moreover, in a study aimed at evaluating the relationship between gut microbiome and the development of type 2 diabetes, Brown et al. reported that high-sensitivity CRP was positively correlated with order Bacteroidales and inversely correlated with order Lachnospirales [39]. The study included a cohort of 616 participants, consisting of 152 with normal glycemia, 368 with prediabetes and 96 with newly identified diabetes. CRP was also positively associated with *Bacteroides* co-abundance group in a study that involved 178 older adult participants [40]. In our study, *B. thetaiotaomicron*, which belongs to order Bacteroidales and *R. gnavus* and *S. intestinalis*, both of which belong to order Lachnospirales, were all positively associated with CRP. We also observed a positive association between purine synthesis in *B. thetaiotaomicron* and CRP when analysis was limited to pathways associated with significant taxa only. *B. thetaiotaomicron*, is an important member of the gut microbiome that was previously reported to regulate immune response through promoting the secretion of IL-10, an anti-inflammatory cytokine [41]. In addition to *B. thetaiotaomicron*, animal studies have shown that *F. plautii* (which was positively associated with CRP in our study) administration has an effect on the regulation of immune response and gut inflammation [42, 43]. The relationship of each of *B. thetaiotaomicron* and *F. plautii* with CRP, however, requires further investigation.

We observed that CRP is inversely associated with gut microbiome alpha diversity (Shannon index). The study by Brown et al. also reported an inverse association between high sensitivity CRP (hsCRP) and gut microbiome alpha diversity (Shannon index) [39]. However, another study on overweight and obese pregnant women did not observe a significant correlation between gut microbiome diversity (Shannon index) and hsCRP levels [44]. Similarly, no association between CRP and gut microbiota diversity (Shannon index) was detected in a study that involved a sample of 115 COVID-19 patients [45]. It should be noted that all of these studies focused on populations with specific traits and had a limited number of participants. CRP is an important downstream inflammatory marker that is known to exhibit both pro-inflammatory and anti-inflammatory functions and is associated with different diseases including cardiovascular disease and cancer [46, 47]. Specific gut microbes can reduce the level of CRP through the release of anti-inflammatory metabolic molecules [48]. One important group of gut metabolites is short-chain fatty acids which play an important role in different processes including the regulation of the immune system [49]. Short-chain fatty acids can reduce the enzymatic synthesis of CRP by the liver [50]. CRP levels could also be lowered due to a decreased expression of IL-6, and it has been reported that probiotic consumption lowers IL-6 expression in ulcerative colitis patients [50, 51].

IL-8 and IP-10 were both linked to alpha diversity. A previous large-scale Mendelian randomization study that used summary statistics from previous genome-wide association studies on microbial taxa and cytokines demonstrated that IL-8 was putatively causally associated with nearly all significant taxa and was also involved in host-taxa interactions [5]. The authors concluded that many taxa influence IL-8. In that study and another, gut microbiome taxa such as phylum Actinobacteria and phylum Euryarchaeota have been reported to be putatively causally associated with levels of IL-8 [5, 52]. In our study, however, no significant associations between IL-8 and common taxa were detected. IL-8 is an inflammatory chemokine that plays an important role in the recruitment and activation of neutrophils to inflammation sites [53]. The gut microbiome has been previously shown to stimulate the secretion of IL-8 by intestinal epithelial cells through the production of butyrate [54]. IP-10, on the other hand, is a chemokine that attracts several types of immune cells and plays a role in stimulating apoptosis, controlling cell growth, and regulating angiogenesis [55]. A previous study has reported a regulatory role of IP-10 in the gut microbiome of rheumatoid arthritis patients [56]. Moreover, alterations in the gut microbiome induce islet-autoimmunity through increasing the production of IP-10 [57]. The association of IL-8 and IP-10 with microbial alpha diversity as discovered in this study, and their links to the gut microbiome observed in prior research, highlight the importance of additional research that focuses on further understanding such associations.

The strengths of our study include the broad panel of cytokines studied (45 cytokines and CRP), the use of shotgun metagenome sequencing, and access to a large, randomly selected population cohort. However, our study should be interpreted within the context of its limitations. Cytokine profiling was performed only for participants above the age of 50. This exclusion both lowered our study sample size and also restricted participants to a certain age group. Despite this, the study sample size remains high compared to previously published studies. Moreover, our study included missing (unmeasured) values for cytokines. However, the majority of the studied cytokines (40/45) had only less than 10% of missing data (unmeasured values), per cytokine. Another limitation is the inability to generalize the results to other cohorts because FINRISK cohort is composed of participants with European or Finnish ancestry.

## Conclusions

In the present work, we detected several significant associations between circulating cytokines and CRP with gut microbiome in a large population-based cohort. We demonstrated that CRP is strongly associated with the gut microbiome as we observed significant results in the majority of the conducted statistical tests. The significant taxa associated with CRP included *B. thetaiotaomicron*, an important resident of the human gut that has been previously linked to the immune response [41]. Functional analysis with pathways associated with significant taxa only showed a positive association between purine synthesis in *B. thetaiotaomicron* and CRP. However, such potential interaction requires further experimental investigation. Another key finding was the association of IL-8 and IP-10 with gut microbiome alpha diversity, which shows the importance of these cytokines in the relationship between the immune system and gut microbiome diversity and composition. Moreover, we observed that MIP-1B, a chemokine whose expression is induced by bacterial endotoxins [29, 30], was associated with specific gut microbiome species and microbial beta diversity. Our study provides key insights into the important relationship of gut microbial diversity and composition with cytokines and CRP. These findings could also lay the groundwork for future experimental studies to verify the potential causality of these associations. Future work should focus on understanding the mechanisms by which the gut microbiome impacts CRP, MIP-1B, and IL-8 expression during diseases development and progression, as targeting this interaction could provide a potential therapeutic approach.

## Supplementary Information

Below is the link to the electronic supplementary material.

Supplementary Material 1

Supplementary Material 2

## Data Availability

The FINRISK data is available through submitting a request to THL biobank (https://thl.fi/en/research-and-development/thl-biobank/for-researchers/application-process). The underlying code for this study is publicly available and can be accessed via this link https://zenodo.org/records/16793433.
