# Supplementary material for: Associations between gut microbiome and circulating cytokines: a cross-sectional analysis in the FINRISK 2002 population cohort
Source: Gut Pathog. 2025 Aug 26;17:66. doi: 10.1186/s13099-025-00742-z (PMC12379326; doi:10.1186/s13099-025-00742-z)
Supplement: Supplementary file 1 — Supplementary Material 1 [file 13099_2025_742_MOESM1_ESM.docx]

**Associations between gut microbiome and circulating cytokines:
a cross-sectional analysis in the FINRISK 2002 population cohort**

**Supplemental Table 1 – Proportion of participants (%) with detectable, above or below detection limits, and unmeasured cytokine and CRP values**

| **Cytokine** | **Detectable cytokines levels** | **No recorded cytokine data** | **Below detection limit** | **Above detection limit** |
| --- | --- | --- | --- | --- |
| MIG | 100.00 | 0.00 | 0.00 | 0.00 |
| IP_10 | 100.00 | 0.00 | 0.00 | 0.00 |
| MCP_1_MCAF | 100.00 | 0.00 | 0.00 | 0.00 |
| PDGF_BB | 100.00 | 0.00 | 0.00 | 0.00 |
| IL_2RA | 99.96 | 0.00 | 0.04 | 0.00 |
| HGF | 99.96 | 0.00 | 0.04 | 0.00 |
| SCGF_B | 99.96 | 0.00 | 0.04 | 0.00 |
| SCF | 99.92 | 0.00 | 0.08 | 0.00 |
| VEGF | 99.92 | 0.00 | 0.08 | 0.00 |
| TRAIL | 99.67 | 0.00 | 0.33 | 0.00 |
| CRP | 99.62 | 0.29 | 0.08 | 0.00 |
| IL_12_P70 | 99.54 | 0.00 | 0.46 | 0.00 |
| IL_18 | 97.66 | 2.34 | 0.00 | 0.00 |
| IL_10 | 97.46 | 0.00 | 2.54 | 0.00 |
| MIP_1B | 97.46 | 2.54 | 0.00 | 0.00 |
| IL_16 | 97.12 | 2.79 | 0.08 | 0.00 |
| CTACK | 97.00 | 2.79 | 0.00 | 0.21 |
| IL_1RA | 96.91 | 2.75 | 0.25 | 0.08 |
| IL_9 | 96.87 | 2.75 | 0.25 | 0.13 |
| M_CSF | 96.25 | 2.34 | 1.42 | 0.00 |
| FGF_BASIC | 95.62 | 2.54 | 1.83 | 0.00 |
| IL_3 | 95.41 | 2.34 | 2.25 | 0.00 |
| IL_6 | 95.00 | 4.88 | 0.13 | 0.00 |
| B_NGF | 94.62 | 5.30 | 0.08 | 0.00 |
| IL_13 | 93.58 | 4.88 | 1.54 | 0.00 |
| IL_12_P40 | 92.37 | 2.79 | 4.84 | 0.00 |
| IL_8 | 92.20 | 7.80 | 0.00 | 0.00 |
| IFN_G | 92.20 | 7.80 | 0.00 | 0.00 |
| GROA | 91.91 | 5.13 | 2.96 | 0.00 |
| IL_4 | 91.87 | 7.80 | 0.33 | 0.00 |
| MIP_1A | 91.78 | 7.80 | 0.42 | 0.00 |
| IL_17 | 91.70 | 7.80 | 0.50 | 0.00 |
| EOTAXIN | 91.41 | 0.00 | 8.59 | 0.00 |
| G_CSF | 91.37 | 8.01 | 0.63 | 0.00 |
| RANTES | 91.33 | 2.79 | 0.00 | 5.88 |
| IL_2 | 88.74 | 5.30 | 5.96 | 0.00 |
| SDF_1A | 87.53 | 5.42 | 7.05 | 0.00 |
| TNF_A | 87.28 | 7.80 | 4.92 | 0.00 |
| MIF | 85.86 | 13.97 | 0.17 | 0.00 |
| TNF_B | 85.36 | 5.30 | 9.34 | 0.00 |
| IL_7 | 83.90 | 16.06 | 0.04 | 0.00 |
| IL_5 | 83.61 | 10.63 | 5.75 | 0.00 |
| IL_1A | 79.52 | 4.88 | 15.60 | 0.00 |
| IL_1B | 79.32 | 10.63 | 10.05 | 0.00 |
| GM_CSF | 66.31 | 18.68 | 15.01 | 0.00 |
| LIF | 60.09 | 0.00 | 39.91 | 0.00 |
| MCP_3 | 49.54 | 21.77 | 28.69 | 0.00 |
| IL_15 | 31.98 | 26.98 | 41.03 | 0.00 |
| IFN_A2 | 14.51 | 84.15 | 1.33 | 0.00 |

**Supplemental Table 2 – Characteristics of study sample stratified by geographical area**

|  | **Eastern Finland** | **Western Finland** |
| --- | --- | --- |
| Characteristic | **Mean (SD) / N (%)** | **Mean (SD) / N (%)** |
| N | 1418 | 980 |
| Age, year (SD) | 60.1 (5.90) | 60.2 (5.97) |
| Men | 676 (47.7%) | 481 (49.1%) |
| BMI (SD) | 28.5 (4.56) | 27.5 (4.35) |
| Diabetes | 105 (7.4%) | 55 (5.6%) |
| Smoking | 209 (14.7%) | 192 (19.6%) |

**Supplemental Table 3 – Mean concentrations of cytokines** (**pg/ml) and CRP (mg/l)**

| **Cytokine** | **Mean** | **SD** | **Number of missing (unmeasured) cytokine values** | **Type^1^** |
| --- | --- | --- | --- | --- |
| MIG | 2026.81 | 2451.73 | 0 | Chemokine |
| EOTAXIN | 66.72 | 227.18 | 0 | Chemokine |
| IP_10 | 598.73 | 563.33 | 0 | Chemokine |
| MCP_1_MCAF | 91.84 | 55.78 | 0 | Chemokine |
| MIP_1B | 71.32 | 62.56 | 61 | Chemokine |
| CTACK | 1983.54 | 688.76 | 67 | Chemokine |
| RANTES | 4422.09 | 4885.73 | 67 | Chemokine |
| GROA | 48.60 | 32.06 | 123 | Chemokine |
| SDF_1A | 80.74 | 66.19 | 130 | Chemokine |
| MIP_1A | 7.00 | 9.40 | 187 | Chemokine |
| HGF | 501.93 | 779.47 | 0 | Growth factor |
| SCF | 280.89 | 106.48 | 0 | Growth factor |
| SCGF_B | 50692.58 | 18890.99 | 0 | Growth factor |
| PDGF_BB | 506.22 | 377.17 | 0 | Growth factor |
| VEGF | 45.96 | 70.81 | 0 | Growth factor |
| M_CSF | 27.47 | 38.86 | 56 | Growth factor |
| FGF_BASIC | 44.54 | 79.81 | 61 | Growth factor |
| B_NGF | 2.46 | 8.00 | 127 | Growth factor |
| G_CSF | 53.66 | 81.34 | 192 | Growth factor |
| IL_7 | 31.07 | 182.11 | 385 | Growth factor |
| GM_CSF | 61.02 | 120.26 | 448 | Growth factor |
| IL_2RA | 258.36 | 159.82 | 0 | Other |
| LIF | 21.16 | 92.04 | 0 | Other |
| TRAIL | 195.72 | 147.33 | 0 | Other |
| IL_10 | 20.14 | 156.33 | 0 | Other |
| IL_12_P70 | 52.57 | 360.95 | 0 | Other |
| CRP | 2.73 | 5.06 | 7 | Other |
| IL_3 | 227.84 | 359.27 | 56 | Other |
| IL_18 | 217.06 | 108.19 | 56 | Other |
| IL_1RA | 593.87 | 6514.34 | 66 | Other |
| IL_9 | 744.39 | 18512.25 | 66 | Other |
| IL_12_P40 | 485.54 | 1135.99 | 67 | Other |
| IL_16 | 390.62 | 241.19 | 67 | Other |
| IL_1A | 2.08 | 3.71 | 117 | Other |
| TNF_B | 14.39 | 16.02 | 127 | Other |
| IL_2 | 16.02 | 108.16 | 127 | Other |
| IL_4 | 2.30 | 11.39 | 187 | Other |
| IL_8 | 13.73 | 22.89 | 187 | Other |
| IL_17 | 65.70 | 290.03 | 187 | Other |
| IFN_G | 167.91 | 1082.27 | 187 | Other |
| TNF_A | 61.91 | 564.27 | 187 | Other |
| IL_1B | 2.77 | 18.90 | 255 | Other |
| IL_5 | 3.82 | 23.29 | 255 | Other |
| MIF | 694.80 | 599.45 | 335 | Other |
| IL_6 | 18.01 | 128.77 | 117 | Other |
| IL_13 | 15.92 | 99.50 | 117 | Other |

SD, standard deviation

^1^ Santalahti, K., Maksimow, M., Airola, A., Pahikkala, T., Hutri-Kähönen, N., Jalkanen, S., Raitakari, O. T., & Salmi, M. (2016). Circulating Cytokines Predict the Development of Insulin Resistance in a Prospective Finnish Population Cohort. *The Journal of clinical endocrinology and metabolism*, *101*(9), 3361–3369. https://doi.org/10.1210/jc.2016-2081

**Supplemental Table 4 –** **Associations of alpha diversity (Shannon index) with cytokines and CRP, calculated using multivariable-adjusted linear regression models. Alpha diversity was defined using Shannon index at species-level. Estimates are reported per 1-SD increment.**

| **Cytokine** | **Estimate** | **Standard error** | **T-value** | **P-value** | **FDR-P** |
| --- | --- | --- | --- | --- | --- |
| IL_8 | -0.066 | 0.021 | -3.155 | 0.002 | 0.025 |
| IP_10 | -0.063 | 0.020 | -3.219 | 0.001 | 0.025 |
| CRP | -0.062 | 0.019 | -3.305 | 0.001 | 0.025 |
| LIF | -0.049 | 0.021 | -2.360 | 0.018 | 0.149 |
| IL_6 | -0.049 | 0.021 | -2.339 | 0.019 | 0.149 |
| IL_10 | -0.049 | 0.021 | -2.390 | 0.017 | 0.149 |
| IL_4 | -0.046 | 0.021 | -2.178 | 0.030 | 0.194 |
| IL_5 | -0.046 | 0.022 | -2.120 | 0.034 | 0.196 |
| EOTAXIN | -0.041 | 0.020 | -2.031 | 0.042 | 0.217 |
| IL_2RA | -0.039 | 0.020 | -1.944 | 0.052 | 0.239 |
| CTACK | 0.037 | 0.020 | 1.843 | 0.066 | 0.258 |
| GROA | -0.038 | 0.021 | -1.812 | 0.070 | 0.258 |
| B_NGF | -0.034 | 0.021 | -1.593 | 0.111 | 0.258 |
| IL_1B | -0.039 | 0.022 | -1.791 | 0.073 | 0.258 |
| IL_9 | -0.034 | 0.021 | -1.635 | 0.102 | 0.258 |
| G_CSF | -0.034 | 0.021 | -1.603 | 0.109 | 0.258 |
| IFN_G | -0.035 | 0.021 | -1.651 | 0.099 | 0.258 |
| MCP_1_MCAF | -0.035 | 0.020 | -1.739 | 0.082 | 0.258 |
| MIP_1A | -0.034 | 0.021 | -1.617 | 0.106 | 0.258 |
| MIP_1B | -0.033 | 0.021 | -1.589 | 0.112 | 0.258 |
| M_CSF | -0.032 | 0.021 | -1.533 | 0.125 | 0.260 |
| MIG | -0.030 | 0.019 | -1.540 | 0.124 | 0.260 |
| IL_12_P70 | -0.031 | 0.021 | -1.515 | 0.130 | 0.260 |
| PDGF_BB | -0.029 | 0.021 | -1.426 | 0.154 | 0.295 |
| VEGF | -0.029 | 0.021 | -1.399 | 0.162 | 0.298 |
| IL_13 | -0.028 | 0.021 | -1.342 | 0.180 | 0.318 |
| GM_CSF | -0.029 | 0.023 | -1.279 | 0.201 | 0.342 |
| SDF_1A | 0.025 | 0.021 | 1.189 | 0.234 | 0.385 |
| TNF_A | -0.023 | 0.021 | -1.080 | 0.280 | 0.444 |
| MIF | 0.022 | 0.023 | 0.980 | 0.327 | 0.502 |
| IL_2 | -0.019 | 0.021 | -0.916 | 0.360 | 0.534 |
| TNF_B | -0.017 | 0.021 | -0.781 | 0.435 | 0.625 |
| IL_1A | 0.014 | 0.021 | 0.680 | 0.496 | 0.640 |
| IL_3 | -0.013 | 0.021 | -0.627 | 0.531 | 0.640 |
| IL_16 | -0.012 | 0.021 | -0.588 | 0.556 | 0.640 |
| IL_18 | -0.012 | 0.020 | -0.605 | 0.545 | 0.640 |
| HGF | -0.013 | 0.020 | -0.662 | 0.508 | 0.640 |
| IL_1RA | -0.015 | 0.021 | -0.694 | 0.487 | 0.640 |
| FGF_BASIC | 0.012 | 0.021 | 0.596 | 0.551 | 0.640 |
| RANTES | -0.014 | 0.021 | -0.671 | 0.502 | 0.640 |
| IL_7 | 0.013 | 0.022 | 0.561 | 0.575 | 0.645 |
| IL_12_P40 | -0.011 | 0.021 | -0.514 | 0.607 | 0.665 |
| TRAIL | -0.008 | 0.021 | -0.386 | 0.700 | 0.748 |
| SCGF_B | -0.005 | 0.020 | -0.250 | 0.803 | 0.839 |
| SCF | 0.004 | 0.020 | 0.198 | 0.843 | 0.854 |
| IL_17 | -0.004 | 0.021 | -0.184 | 0.854 | 0.854 |

FDR, false discovery rate

**Supplemental Table 5 –** **Associations of Principal Component Analysis (PCA) axes with cytokines and CRP, calculated using multivariable-adjusted linear regression models. Estimates are reported per 1-SD increment.**

| **Cytokines** | **Axis** | **Estimate** | **Standard error** | **T-value** | **P-value** | **FDR-P** |
| --- | --- | --- | --- | --- | --- | --- |
| MIP_1B | PC2 | -0.082 | 0.021 | -3.925 | 0.0001 | 0.008 |
| CRP | PC1 | -0.071 | 0.019 | -3.723 | 0.0002 | 0.008 |
| MIG | PC2 | -0.071 | 0.019 | -3.645 | 0.0003 | 0.008 |
| CRP | PC2 | -0.056 | 0.019 | -2.927 | 0.003 | 0.080 |
| TRAIL | PC2 | 0.058 | 0.021 | 2.788 | 0.005 | 0.098 |
| MIP_1B | PC1 | -0.054 | 0.021 | -2.615 | 0.009 | 0.138 |
| IL_18 | PC1 | -0.052 | 0.020 | -2.527 | 0.012 | 0.152 |
| IL_4 | PC2 | -0.042 | 0.022 | -1.923 | 0.055 | 0.628 |
| IL_8 | PC1 | -0.040 | 0.021 | -1.864 | 0.062 | 0.638 |
| CTACK | PC2 | -0.036 | 0.020 | -1.768 | 0.077 | 0.645 |
| IL_8 | PC2 | -0.039 | 0.022 | -1.780 | 0.075 | 0.645 |
| B_NGF | PC2 | -0.036 | 0.021 | -1.668 | 0.096 | 0.676 |
| IFN_G | PC2 | -0.037 | 0.022 | -1.702 | 0.089 | 0.676 |
| VEGF | PC1 | -0.033 | 0.021 | -1.577 | 0.115 | 0.755 |
| IL_3 | PC1 | 0.024 | 0.021 | 1.161 | 0.246 | 0.759 |
| GROA | PC1 | -0.025 | 0.021 | -1.193 | 0.233 | 0.759 |
| LIF | PC1 | -0.019 | 0.021 | -0.933 | 0.351 | 0.759 |
| MIF | PC1 | 0.028 | 0.022 | 1.272 | 0.203 | 0.759 |
| B_NGF | PC1 | 0.028 | 0.021 | 1.311 | 0.190 | 0.759 |
| SCF | PC1 | 0.027 | 0.021 | 1.306 | 0.192 | 0.759 |
| SDF_1A | PC1 | 0.029 | 0.021 | 1.386 | 0.166 | 0.759 |
| TRAIL | PC1 | 0.018 | 0.021 | 0.881 | 0.379 | 0.759 |
| IL_2 | PC1 | -0.022 | 0.021 | -1.013 | 0.311 | 0.759 |
| IL_6 | PC1 | -0.031 | 0.021 | -1.486 | 0.137 | 0.759 |
| IL_7 | PC1 | 0.020 | 0.023 | 0.872 | 0.383 | 0.759 |
| IL_9 | PC1 | -0.018 | 0.021 | -0.857 | 0.392 | 0.759 |
| IL_12_P70 | PC1 | -0.017 | 0.021 | -0.798 | 0.425 | 0.759 |
| IL_17 | PC1 | -0.022 | 0.022 | -1.028 | 0.304 | 0.759 |
| EOTAXIN | PC1 | -0.016 | 0.021 | -0.785 | 0.433 | 0.759 |
| G_CSF | PC1 | -0.020 | 0.022 | -0.913 | 0.361 | 0.759 |
| IFN_G | PC1 | -0.018 | 0.022 | -0.828 | 0.408 | 0.759 |
| IP_10 | PC1 | -0.025 | 0.020 | -1.243 | 0.214 | 0.759 |
| MIP_1A | PC1 | -0.027 | 0.022 | -1.263 | 0.207 | 0.759 |
| PDGF_BB | PC1 | -0.029 | 0.021 | -1.407 | 0.160 | 0.759 |
| IL_2RA | PC2 | -0.021 | 0.021 | -1.005 | 0.315 | 0.759 |
| IL_3 | PC2 | -0.018 | 0.021 | -0.836 | 0.403 | 0.759 |
| IL_12_P40 | PC2 | 0.026 | 0.021 | 1.238 | 0.216 | 0.759 |
| IL_18 | PC2 | -0.022 | 0.021 | -1.065 | 0.287 | 0.759 |
| LIF | PC2 | 0.021 | 0.021 | 0.980 | 0.327 | 0.759 |
| SCF | PC2 | -0.022 | 0.021 | -1.054 | 0.292 | 0.759 |
| SCGF_B | PC2 | -0.017 | 0.021 | -0.798 | 0.425 | 0.759 |
| IL_1B | PC2 | 0.026 | 0.022 | 1.170 | 0.242 | 0.759 |
| IL_2 | PC2 | -0.027 | 0.022 | -1.250 | 0.212 | 0.759 |
| IL_6 | PC2 | -0.031 | 0.021 | -1.454 | 0.146 | 0.759 |
| IL_10 | PC2 | -0.016 | 0.021 | -0.777 | 0.437 | 0.759 |
| IL_12_P70 | PC2 | -0.023 | 0.021 | -1.118 | 0.264 | 0.759 |
| IL_17 | PC2 | -0.024 | 0.022 | -1.101 | 0.271 | 0.759 |
| EOTAXIN | PC2 | -0.022 | 0.021 | -1.051 | 0.293 | 0.759 |
| G_CSF | PC2 | -0.020 | 0.022 | -0.921 | 0.357 | 0.759 |
| MIP_1A | PC2 | 0.018 | 0.022 | 0.829 | 0.407 | 0.759 |
| RANTES | PC2 | -0.018 | 0.021 | -0.848 | 0.397 | 0.759 |
| TNF_A | PC2 | -0.018 | 0.022 | -0.810 | 0.418 | 0.759 |
| VEGF | PC2 | -0.029 | 0.021 | -1.389 | 0.165 | 0.759 |
| MIF | PC2 | 0.016 | 0.022 | 0.738 | 0.461 | 0.785 |
| IL_1A | PC1 | 0.014 | 0.021 | 0.666 | 0.506 | 0.817 |
| HGF | PC2 | -0.013 | 0.020 | -0.658 | 0.511 | 0.817 |
| IL_7 | PC2 | -0.015 | 0.023 | -0.651 | 0.515 | 0.817 |
| PDGF_BB | PC2 | -0.014 | 0.021 | -0.655 | 0.513 | 0.817 |
| FGF_BASIC | PC2 | -0.013 | 0.021 | -0.616 | 0.538 | 0.839 |
| IL_10 | PC1 | -0.012 | 0.021 | -0.587 | 0.557 | 0.854 |
| IL_2RA | PC1 | -0.008 | 0.020 | -0.395 | 0.693 | 0.886 |
| IL_12_P40 | PC1 | 0.008 | 0.021 | 0.402 | 0.687 | 0.886 |
| IL_16 | PC1 | -0.008 | 0.021 | -0.387 | 0.699 | 0.886 |
| HGF | PC1 | -0.008 | 0.020 | -0.424 | 0.672 | 0.886 |
| M_CSF | PC1 | -0.008 | 0.021 | -0.381 | 0.703 | 0.886 |
| GM_CSF | PC1 | -0.011 | 0.023 | -0.479 | 0.632 | 0.886 |
| RANTES | PC1 | 0.009 | 0.021 | 0.428 | 0.669 | 0.886 |
| TNF_A | PC1 | 0.010 | 0.022 | 0.454 | 0.650 | 0.886 |
| IL_16 | PC2 | 0.011 | 0.021 | 0.517 | 0.605 | 0.886 |
| IL_1RA | PC2 | -0.008 | 0.021 | -0.381 | 0.703 | 0.886 |
| IL_5 | PC2 | -0.012 | 0.022 | -0.518 | 0.604 | 0.886 |
| IL_9 | PC2 | 0.008 | 0.021 | 0.386 | 0.700 | 0.886 |
| MCP_1_MCAF | PC2 | -0.008 | 0.021 | -0.385 | 0.701 | 0.886 |
| IP_10 | PC2 | -0.007 | 0.020 | -0.366 | 0.714 | 0.888 |
| MIG | PC1 | 0.005 | 0.019 | 0.275 | 0.783 | 0.925 |
| IL_4 | PC1 | -0.006 | 0.022 | -0.279 | 0.780 | 0.925 |
| GROA | PC2 | -0.007 | 0.022 | -0.303 | 0.762 | 0.925 |
| SDF_1A | PC2 | -0.006 | 0.021 | -0.268 | 0.788 | 0.925 |
| IL_13 | PC2 | -0.006 | 0.022 | -0.261 | 0.794 | 0.925 |
| SCGF_B | PC1 | 0.004 | 0.021 | 0.182 | 0.856 | 0.930 |
| TNF_B | PC1 | 0.003 | 0.021 | 0.152 | 0.879 | 0.930 |
| IL_1B | PC1 | 0.003 | 0.022 | 0.156 | 0.876 | 0.930 |
| IL_1RA | PC1 | -0.004 | 0.021 | -0.193 | 0.847 | 0.930 |
| IL_13 | PC1 | -0.004 | 0.021 | -0.196 | 0.845 | 0.930 |
| FGF_BASIC | PC1 | 0.004 | 0.021 | 0.190 | 0.850 | 0.930 |
| IL_1A | PC2 | 0.005 | 0.022 | 0.228 | 0.820 | 0.930 |
| GM_CSF | PC2 | -0.004 | 0.023 | -0.152 | 0.880 | 0.930 |
| IL_5 | PC1 | -0.003 | 0.022 | -0.139 | 0.890 | 0.930 |
| MCP_1_MCAF | PC1 | 0.002 | 0.021 | 0.096 | 0.924 | 0.955 |
| TNF_B | PC2 | 0.002 | 0.022 | 0.071 | 0.943 | 0.964 |
| CTACK | PC1 | 0.000 | 0.020 | -0.012 | 0.991 | 0.991 |
| M_CSF | PC2 | 0.000 | 0.021 | 0.013 | 0.989 | 0.991 |

FDR, false discovery rate

**Supplemental Figure 1. Top taxa contributing to the first two principal axes. The top ten species with the largest loadings are shown and colored according to their class. The indicated species explain most variation along the first two PCA axes.**


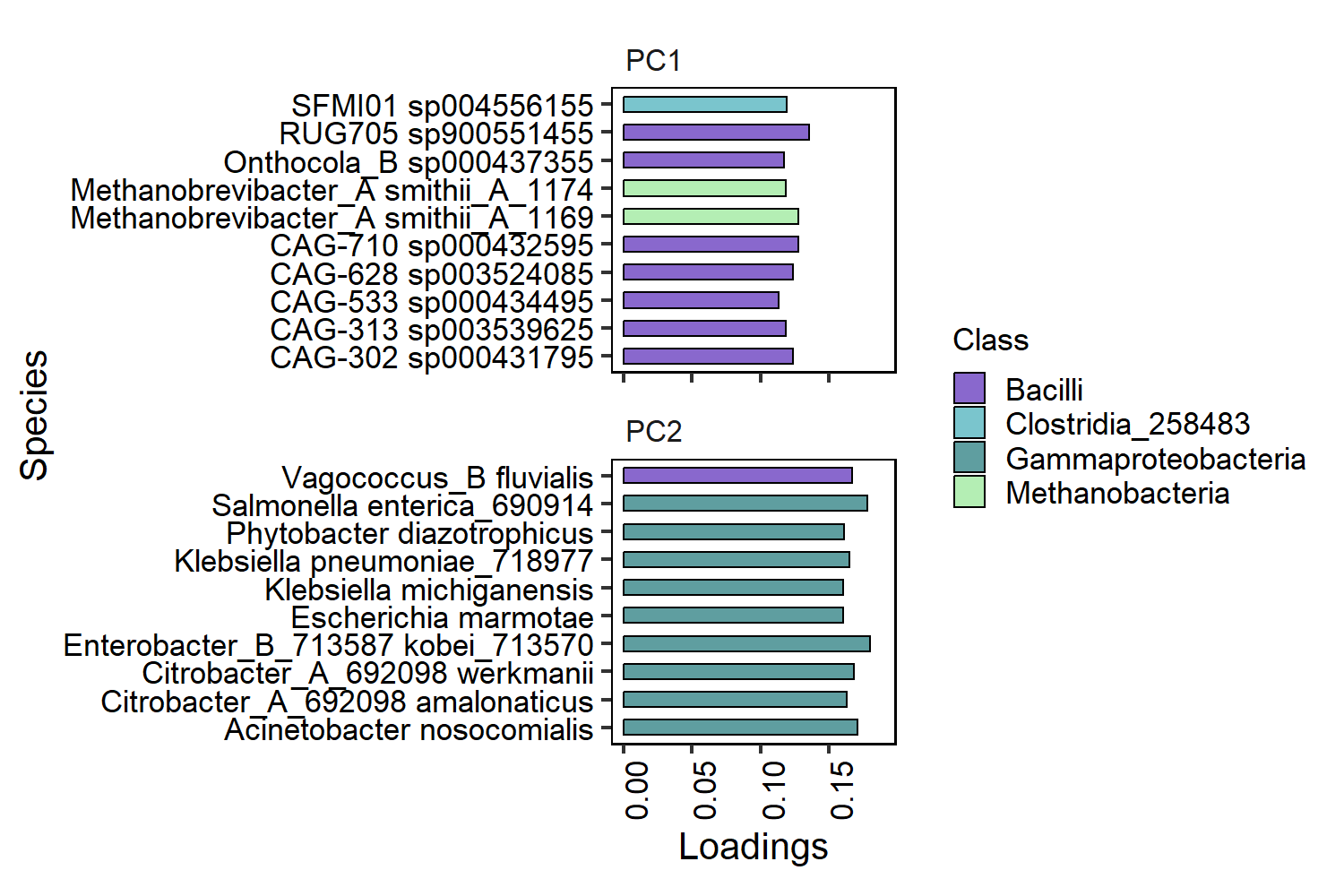


PC, Principal component

**Supplemental Figure 2. Top 25 species-cytokines (and CRP) associations identified using multivariable-adjusted linear regression models. The species that were significantly associated with cytokines/CRP (FDR<0.05) are marked with an asterisk (*). Estimates are reported per 1-SD increment.**


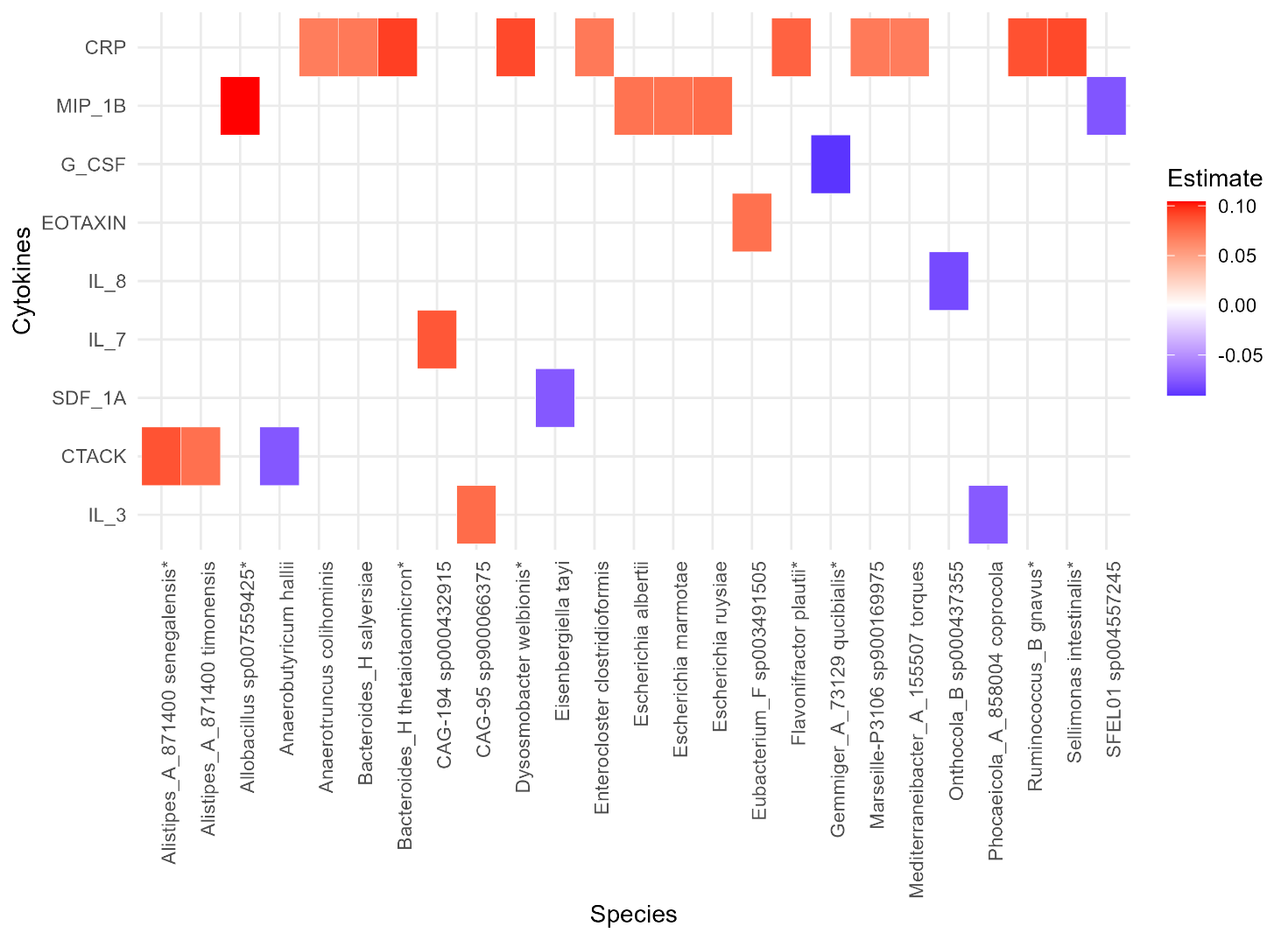


CRP, C-reactive protein; MIP_1B, macrophage inflammatory protein-1β; G_CSF, granulocyte colony-stimulating factor; IL_8, interleukin-8; IL_7, interleukin-7; SDF_1A, stromal derived factor-1α; CTACK, cutaneous T cell-attracting chemokine; IL_3, interleukin-3

**Supplemental Figure 3. *B. thetaiotaomicron* pathways associated with CRP and detected using multivariable-adjusted linear regression models with dichotomized or inverse-rank normalized count data. Estimates are reported per 1-SD increment.**


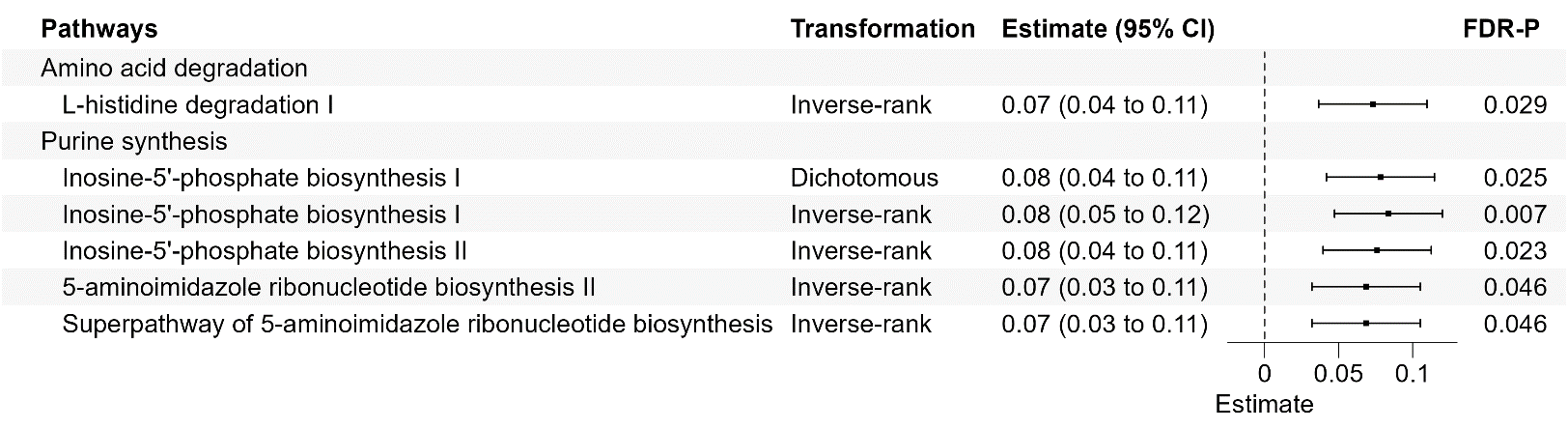


CI, confidence interval; FDR, false discovery rate
